# Supplementary material for: BinSPreader: Refine binning results for fuller MAG reconstruction
Source: iScience. 2022 Jul 19;25(8):104770. doi: 10.1016/j.isci.2022.104770 (PMC9386100; doi:10.1016/j.isci.2022.104770)
Supplement: Document S1. Figures S1–S15 and Tables S1–S17 [file mmc1.pdf]

iScience, Volume *25*

Supplemental information

BinSPreader: Refine binning results

for fuller MAG reconstruction

Ivan Tolstoganov, Yuri Kamenev, Roman Kruglikov, Sofia Ochkalova, and Anton Korobeynikov

Supplementary materials to "BinSPreader: refine binning results for fuller MAG reconstruction"

July 11, 2022

1 Command lines

- Binnacle

```
minimap2 -ax sr <assembly.fasta> <pe_reads1.fq> <pe_reads2.fq> |
samtools view -F 3584 -b -o - |
samtools sort -o <alignment.bam>

MetaCarvel/run.py -a <assembly.fasta> -m <alignment.bam> -d <metacarvel_output>
-k true -r true

binnacle/src/Estimate_Abundances.py -g <metacarvel_output/oriented.gml>
-bam <alignment.bam> -c <assembly.fasta> -d <binnacle_output>

binnacle/src/Collate.py -d <binnacle_output> -m metabat

metabat2 -i <binnacle_output>/Scaffolds.fasta -o <binnacle_output/bins>
-a <binnacle_output/Feature-Matrix-metabat.txt> --seed 42
```

- BinSPreader

```
bin-refine <assembly_graph.gfa> <initial_binning.tsv> <output> -Rcorr -Smle
```

- BinSPreader-PE and BinSPreader-HiC

```
bin-refine <assembly_graph.gfa> <initial_binning.tsv> <output>
--dataset <paired-end or Hi-C library.yaml> -Rcorr -Smle
```

- BinSPreader-Multiple

```
bin-refine <assembly_graph.gfa> <initial_binning.tsv> <output> -Rcorr -Smle -m
```

- DASTool

```
DAS_Tool -i <initial_binning.tsv>  -o <dastool_output>
--search_engine diamond -c <assembly.fasta> --write_bins
```

- MetaBAT2

```
minimap2 -ax sr <assembly.fasta> <pe_reads1.fq> <pe_reads2.fq> |
samtools view -F 3584 -b -o - |
samtools sort -o <alignment.bam>

jgi_summarize_bam_contig_depths --outputDepth <jgi_abundancies> <alignment.bam>

metabat2 -i <assembly.fasta> -o <metabat_output>
-a <jgi_abundancies> --seed 42
```

- MetaCoAG

```
MetaCoAG --assembler SPAdes --graph <assembly_graph.gfa>
--abundance <abundance.tsv> --contigs <assembly.fasta>
--output <output> --paths <assembly.paths> --depth 10
```

- METAMVGL

```
minimap2 -ax sr <assembly.fasta> <pe_reads1.fq> <pe_reads2.fq> |
samtools view -F 3584 -b -o - |
samtools sort -o <alignment.bam>

METAMVGL/prepResult.py --binned <initial_binning_output>
            --assembler SPAdes --output <metamvgl_output>

METAMVGL/prep_graph --assembly-graph <assembly_graph.fastg>
            --bam <alignment.bam> --assembler metaSPAdes
            --output <scaffold_graph> --paths <assembly.paths>

METAMVGL.py --contigs <assembly.fasta> --assembler metaSPAdes
            --assembly_graph <scaffold_graph.ag> --PE_graph <scaffold_graph.pe>
            --binned <initial_binning.csv> --output <metamvgl_output/bins>
```

- MetaWRAP

```
metawrap binning -o <output> -a <assembly.fasta>
--metabat2 --maxbin2 --concoct
```

- VAMB

```
vamb --outdir <output> --fasta <assembly.fasta> --jgi <abundance.depth>
--minfasta 500000 -m 2000
```

2 Supplementary Figures

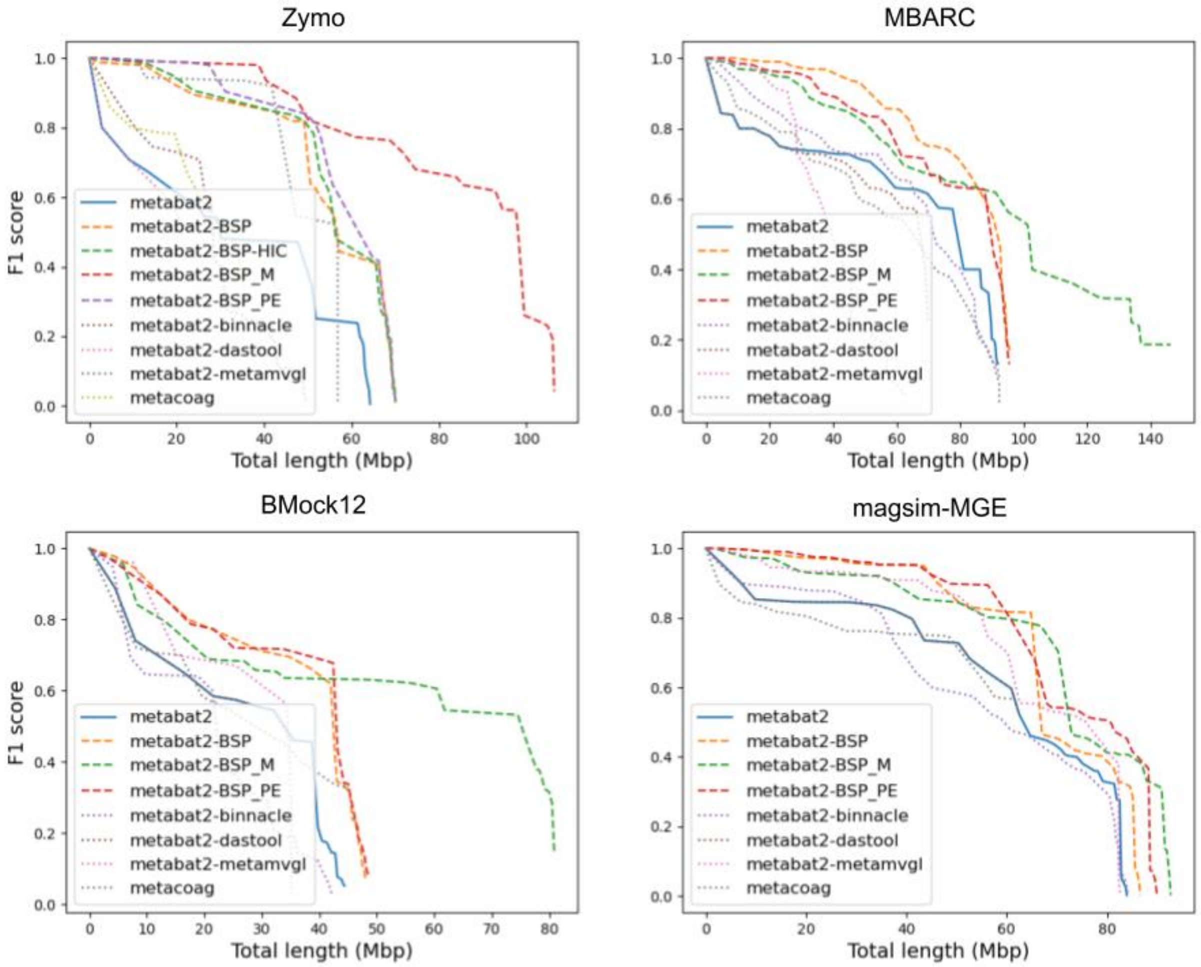

Supplementary Figure 1: F1 score and total length of MAGs for Zymo (top left), MBARC (top right), BMock12 (bottom left), and magsim-MGE (bottom right) datasets, arranged by descending order of F1 (seq) score reported by AMBER, related to Figure 1. Initial binning was produced using metaBAT2 (solid line), refined binnings were produced using DAS_TOOL, Binnacle, and METAMVGL (dotted lines), and three different modes of BinSPreader (dashed lines).

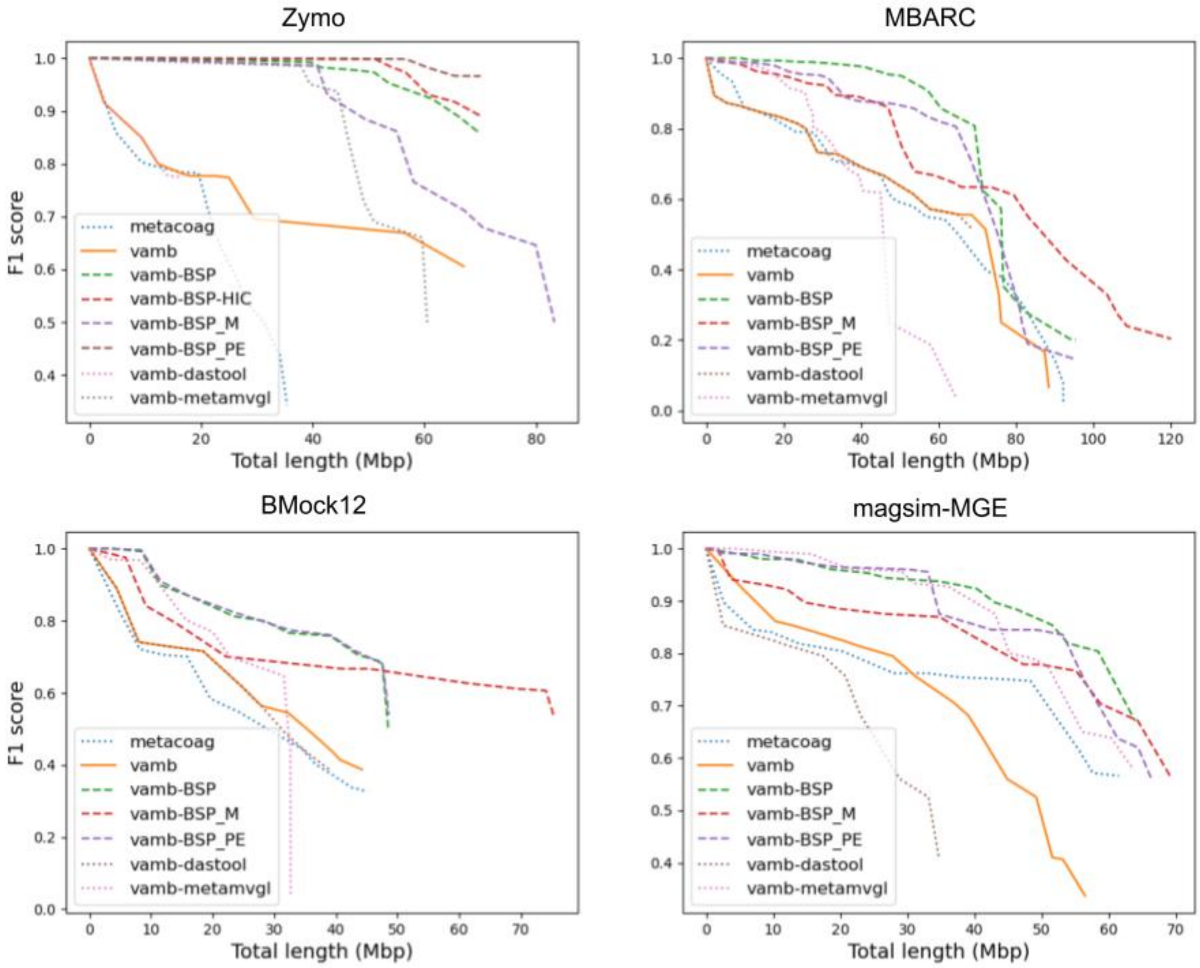

Supplementary Figure 2: F1 score and total length of MAGs for Zymo (top left), MBARC (top right), BMock12 (bottom left), and magsim-MGE (bottom right) datasets, arranged by descending order of F1 (seq) score reported by AMBER, related to Figure 1. Initial binning was produced using VAMB (solid line), refined binnings were produced using DAS_TOOL and METAMVGL (dotted lines), and three different modes of BinSPreader (dashed lines).

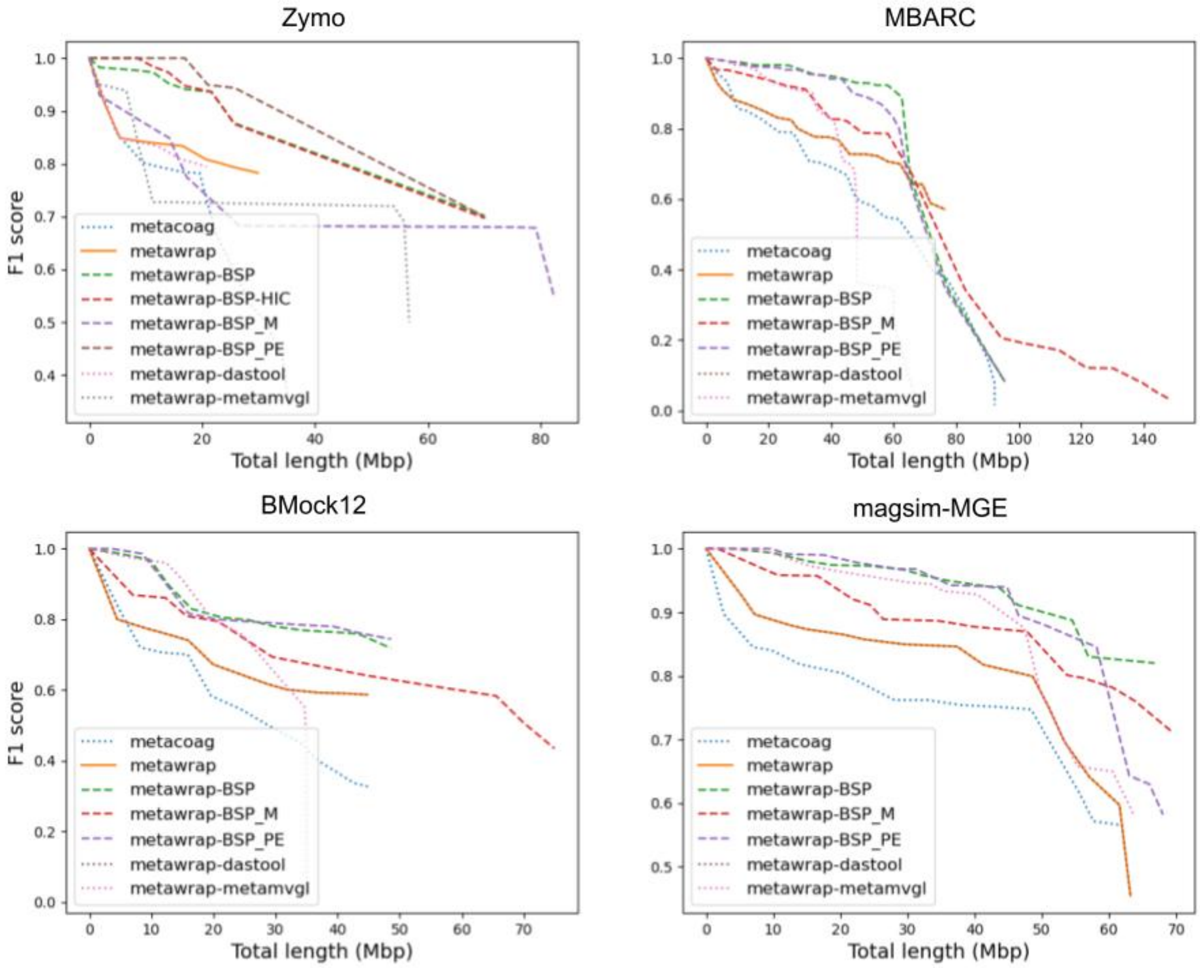

Supplementary Figure 3: F1 score and total length of MAGs for Zymo (top left), MBARC (top right), BMock12 (bottom left), and magsim-MGE (bottom right) datasets, arranged by descending order of F1 (seq) score reported by AMBER, related to Figure 1. Initial binning was produced using MetaWRAP (solid line), refined binnings were produced using DAS_TOOL and METAMVGL (dotted lines), and three different modes of BinSPreader (dashed lines).

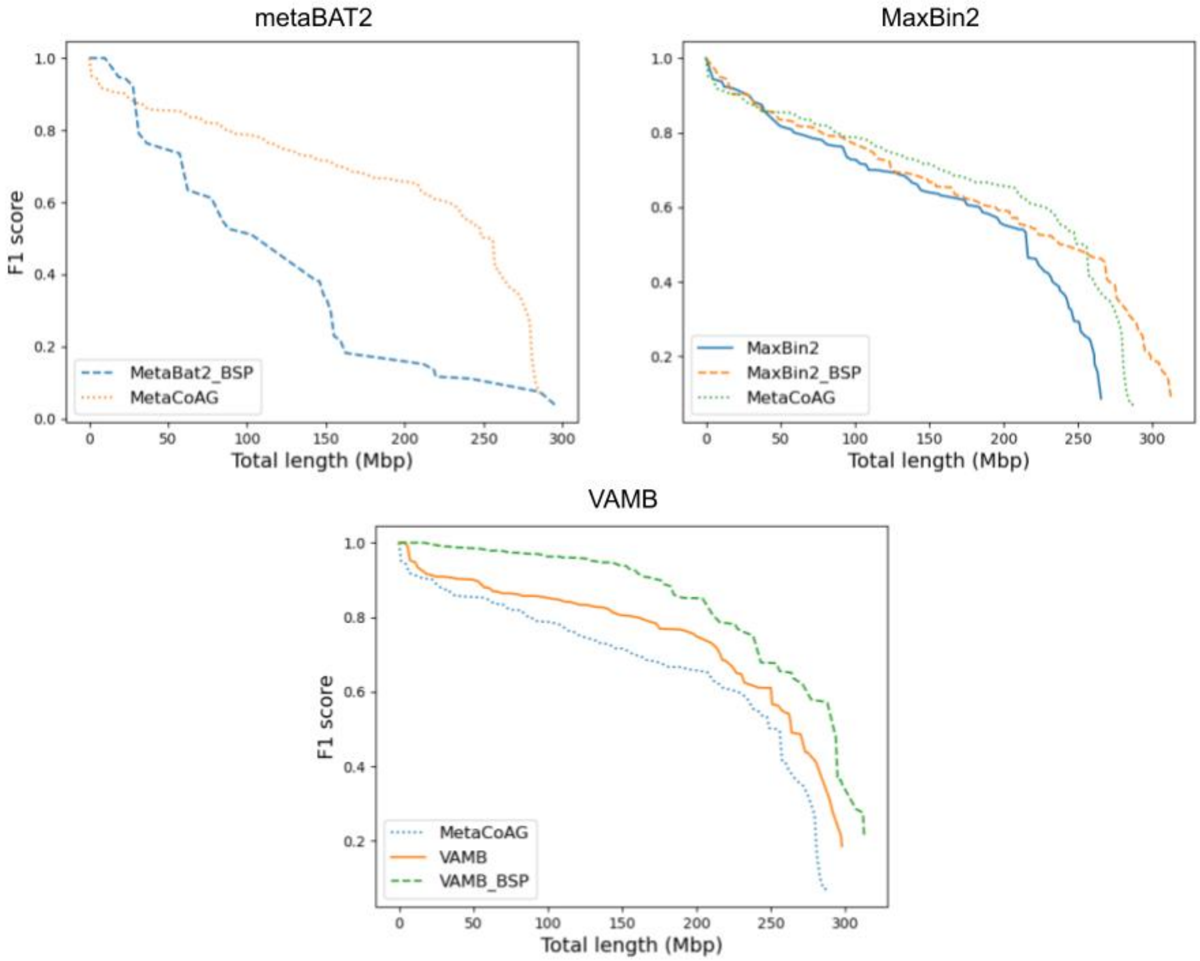

Supplementary Figure 4: F1 score and total length of MAGs for simHC+ dataset, arranged by descending order of F1 (seq) score reported by AMBER, related to Figure 1. Initial binning was produced using MetaWRAP (top left), MaxBin2 (top right), and VAMB (bottom). Refined binnings were produced using BinSPreader (dashed line) and MetaCoAG (dotted line).

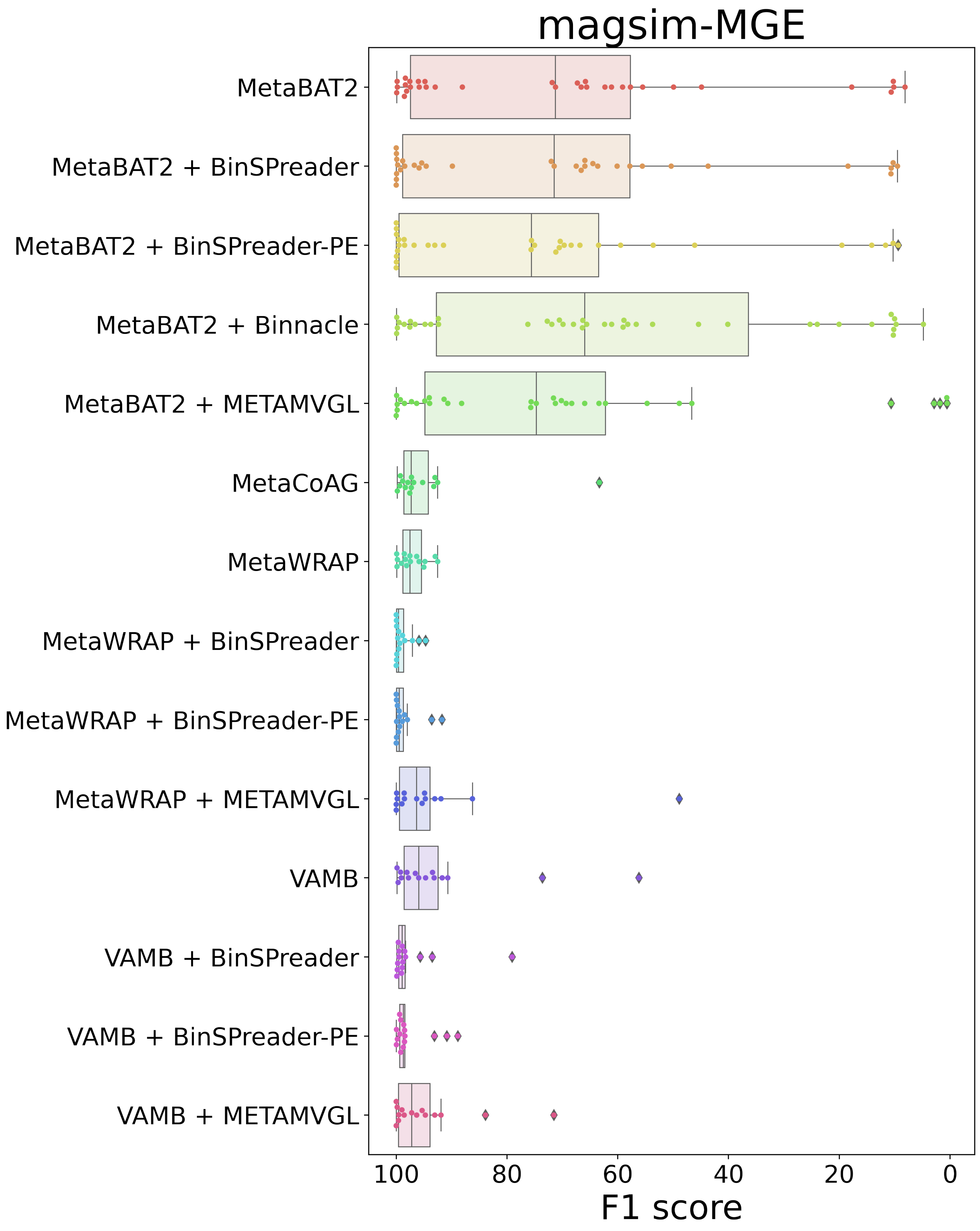

Supplementary Figure 5: F1 score distributions of **magsim-MGE** bins, related to Figure 1.

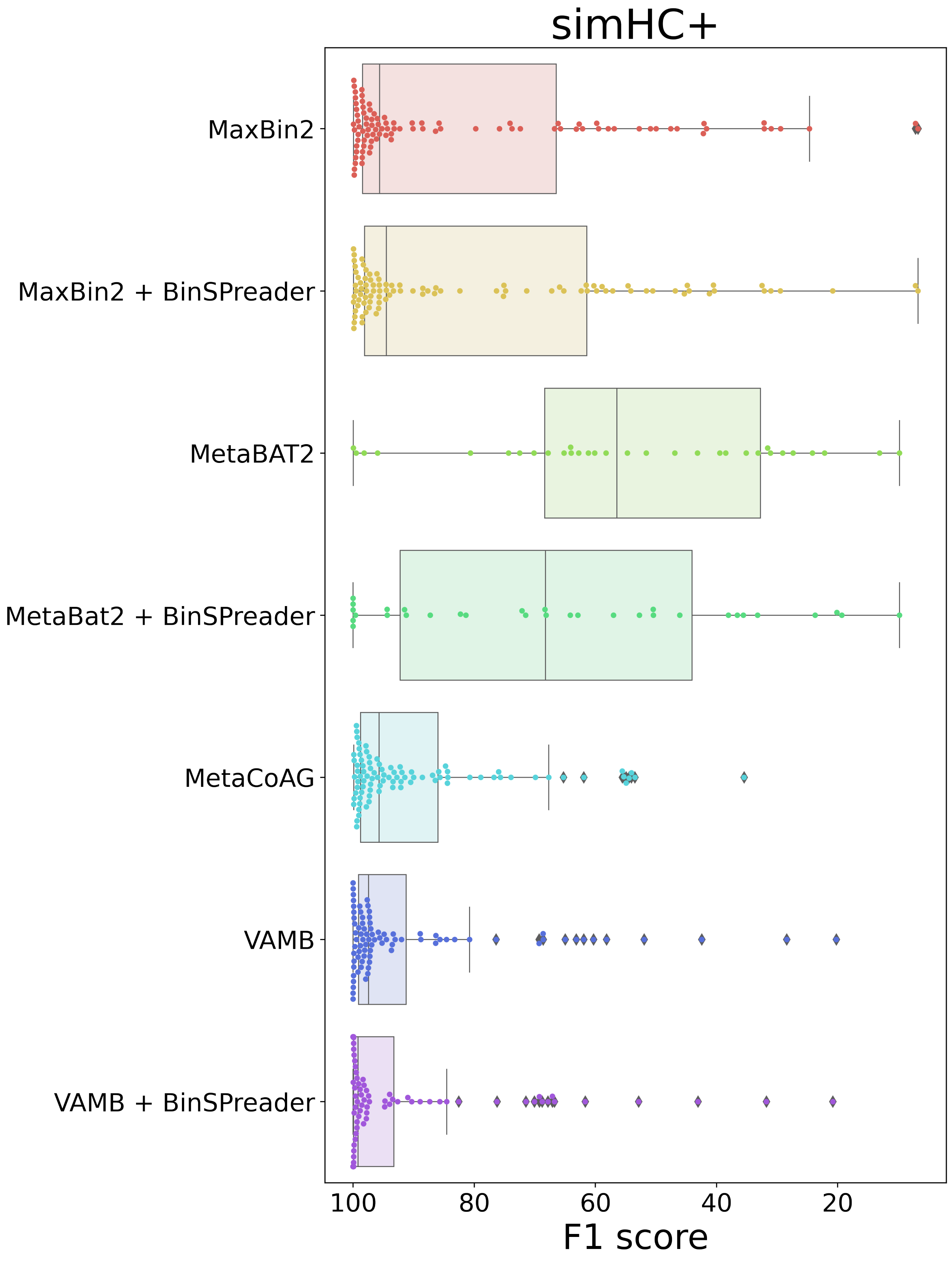

Supplementary Figure 6: F1 score distributions of **simHC+** bins, related to Figure 1.

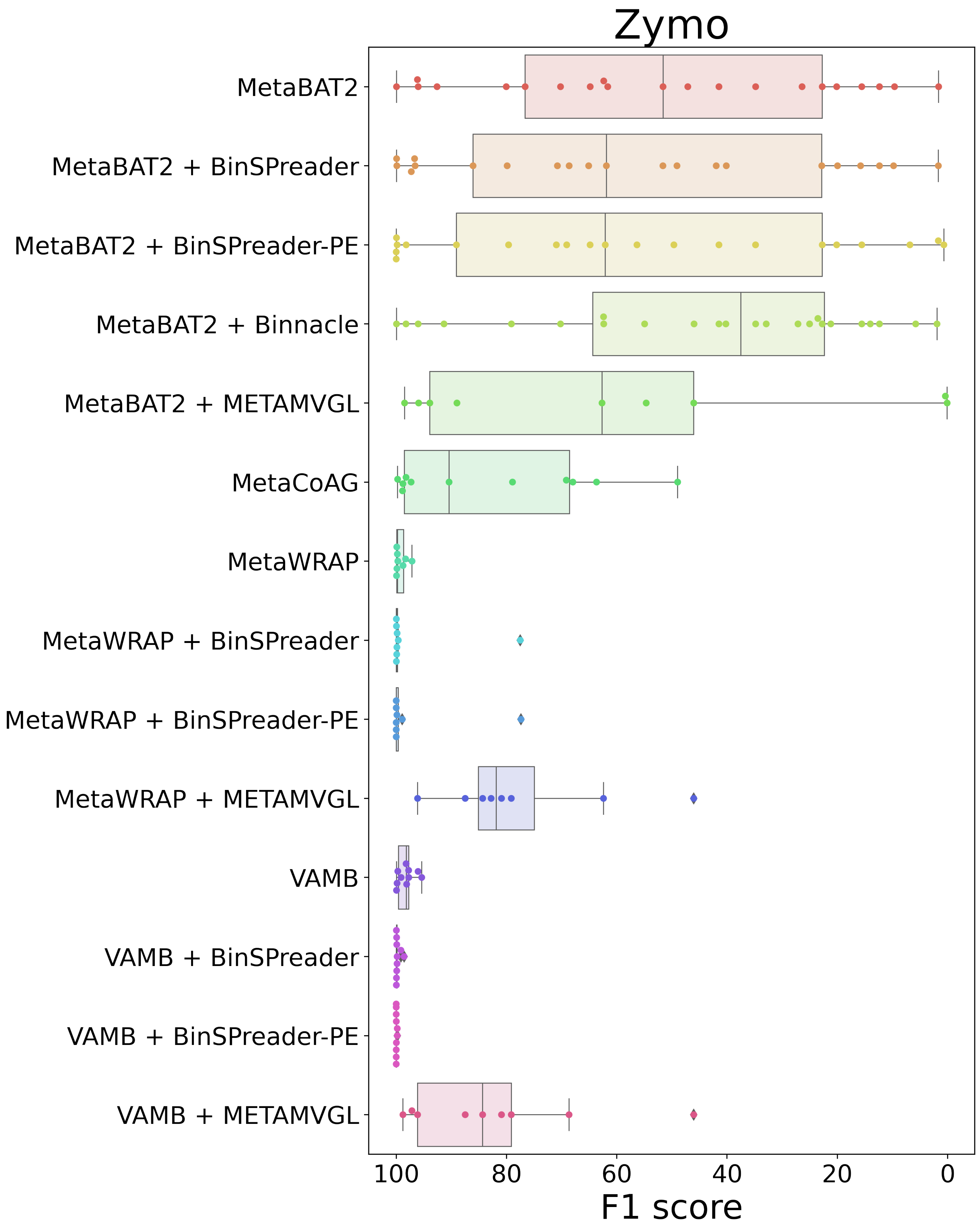

Supplementary Figure 7: F1 score distributions of **Zymo** dataset, related to Figure 1.

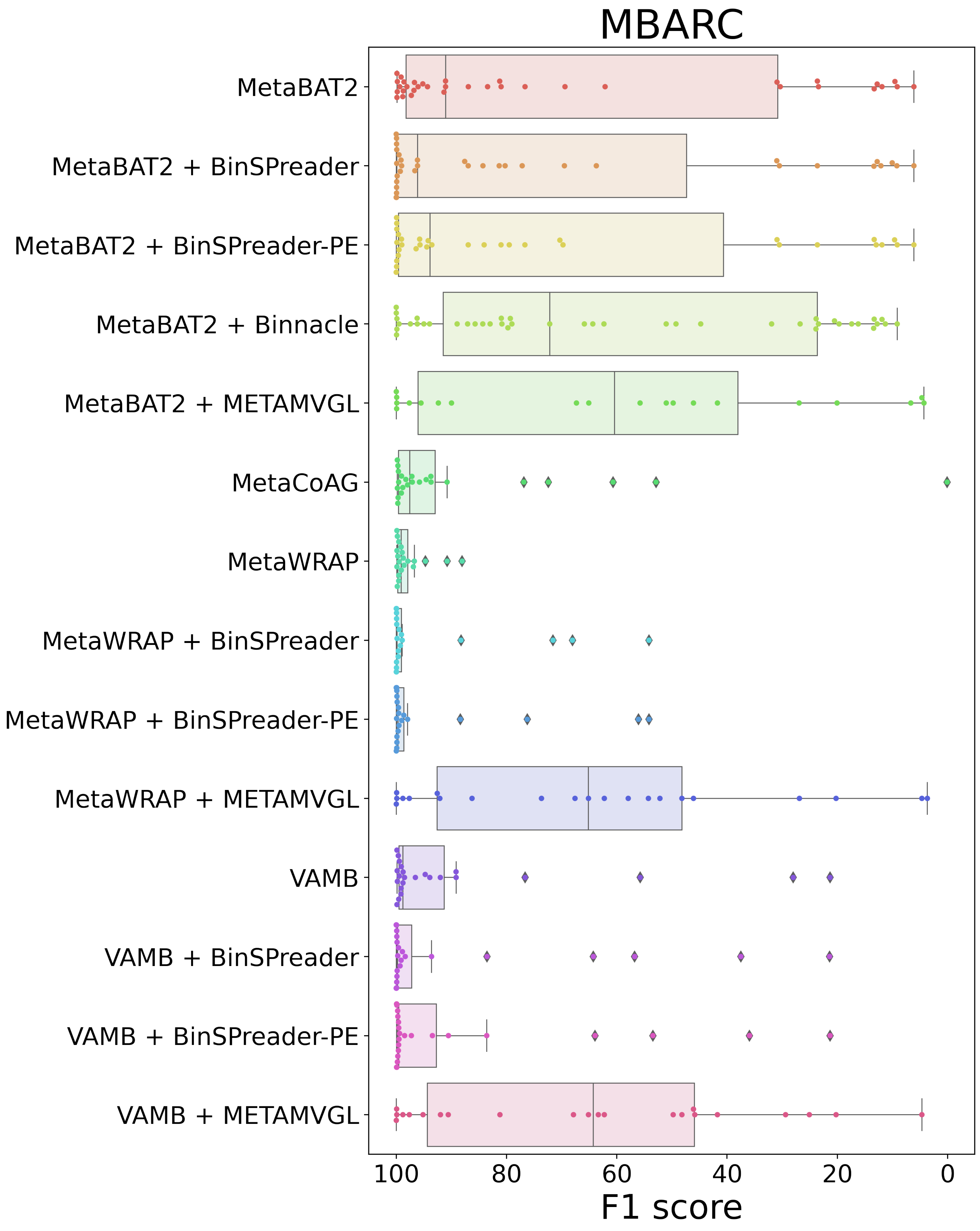

Supplementary Figure 8: F1 score distributions of **MBARC26** bins, related to Figure 1.

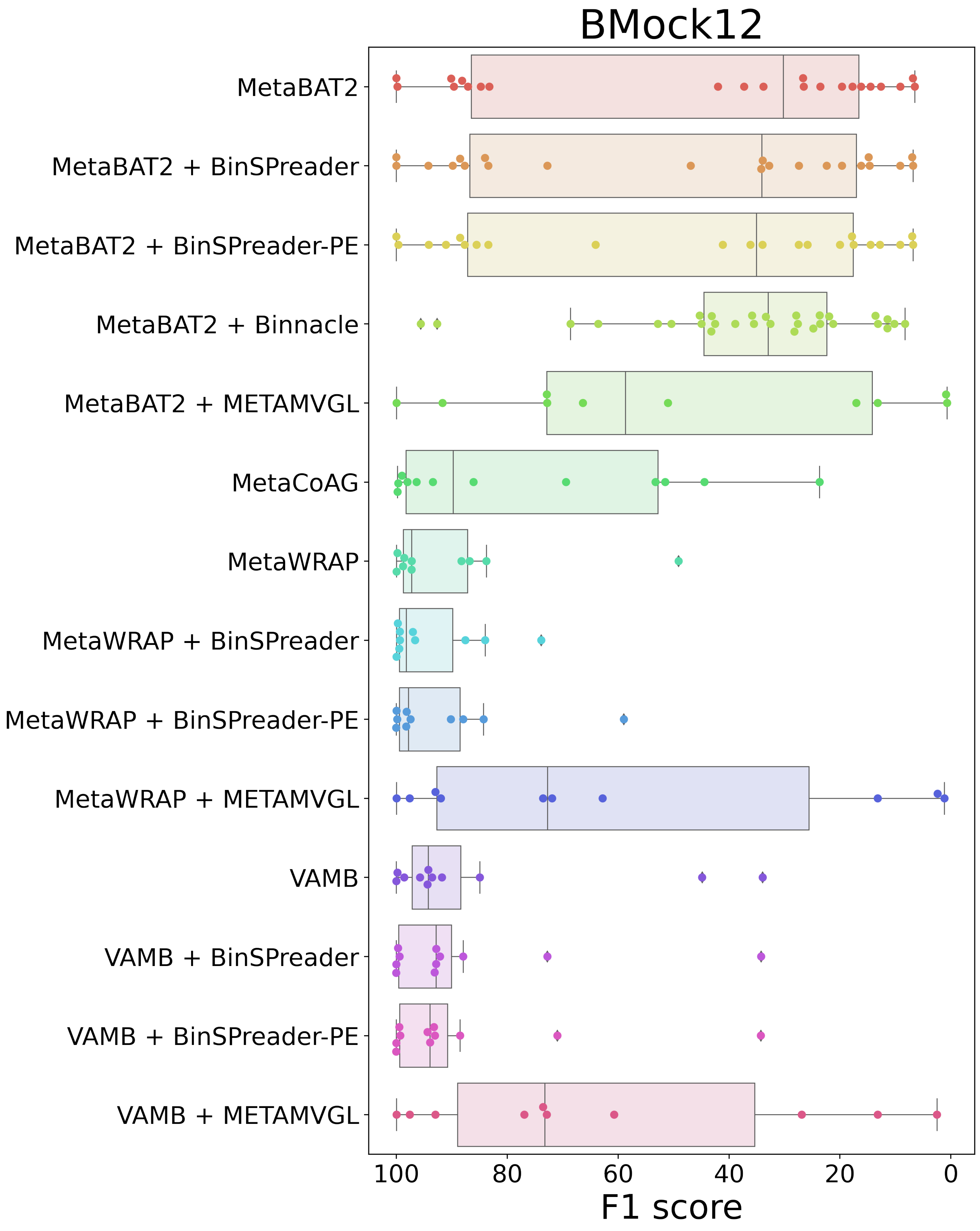

Supplementary Figure 9: F1 score distributions of **BMock12** bins, related to Figure 1.

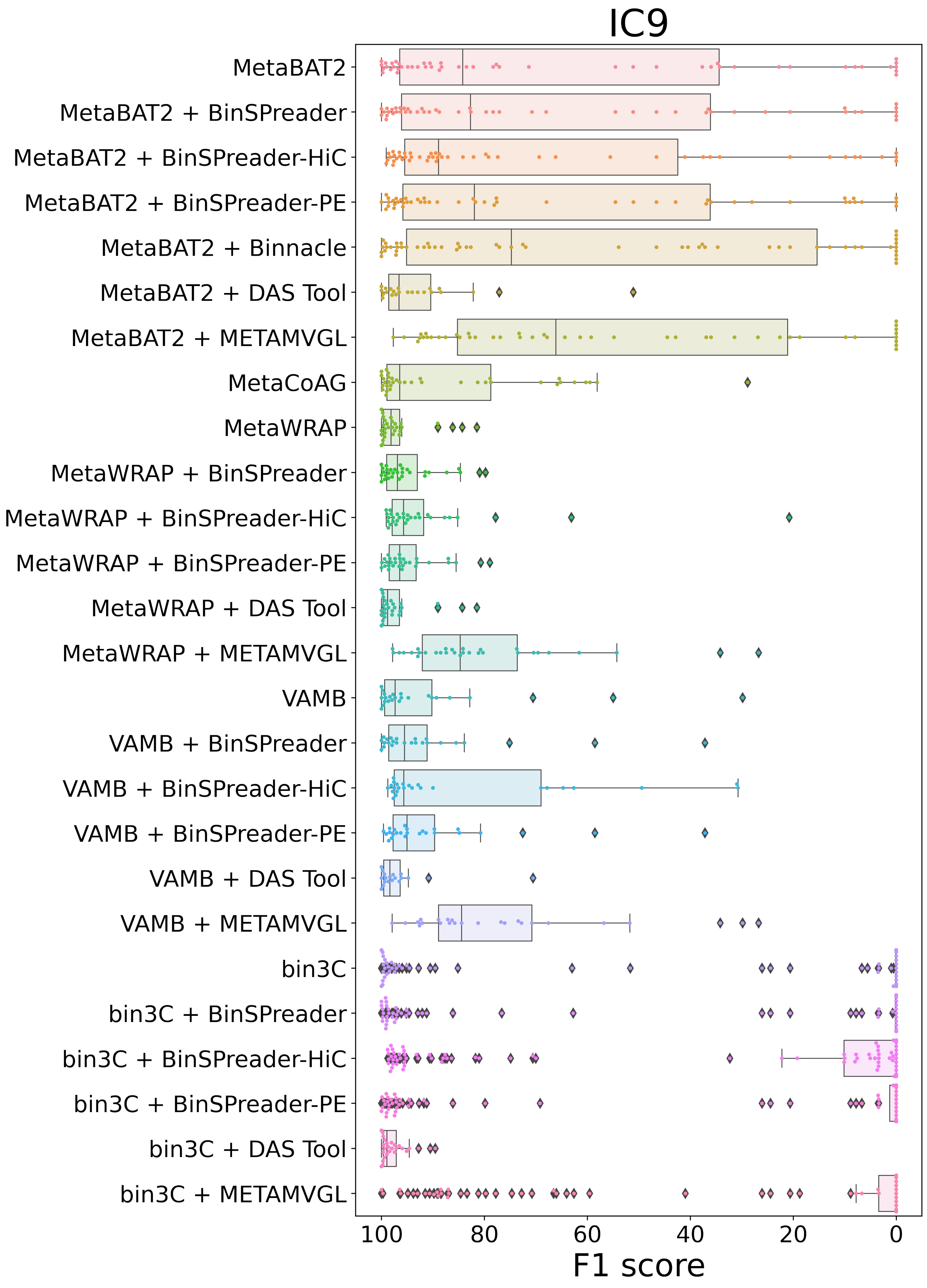

Supplementary Figure 10: F1 score distributions of **IC9** bins, related to Figure 1.

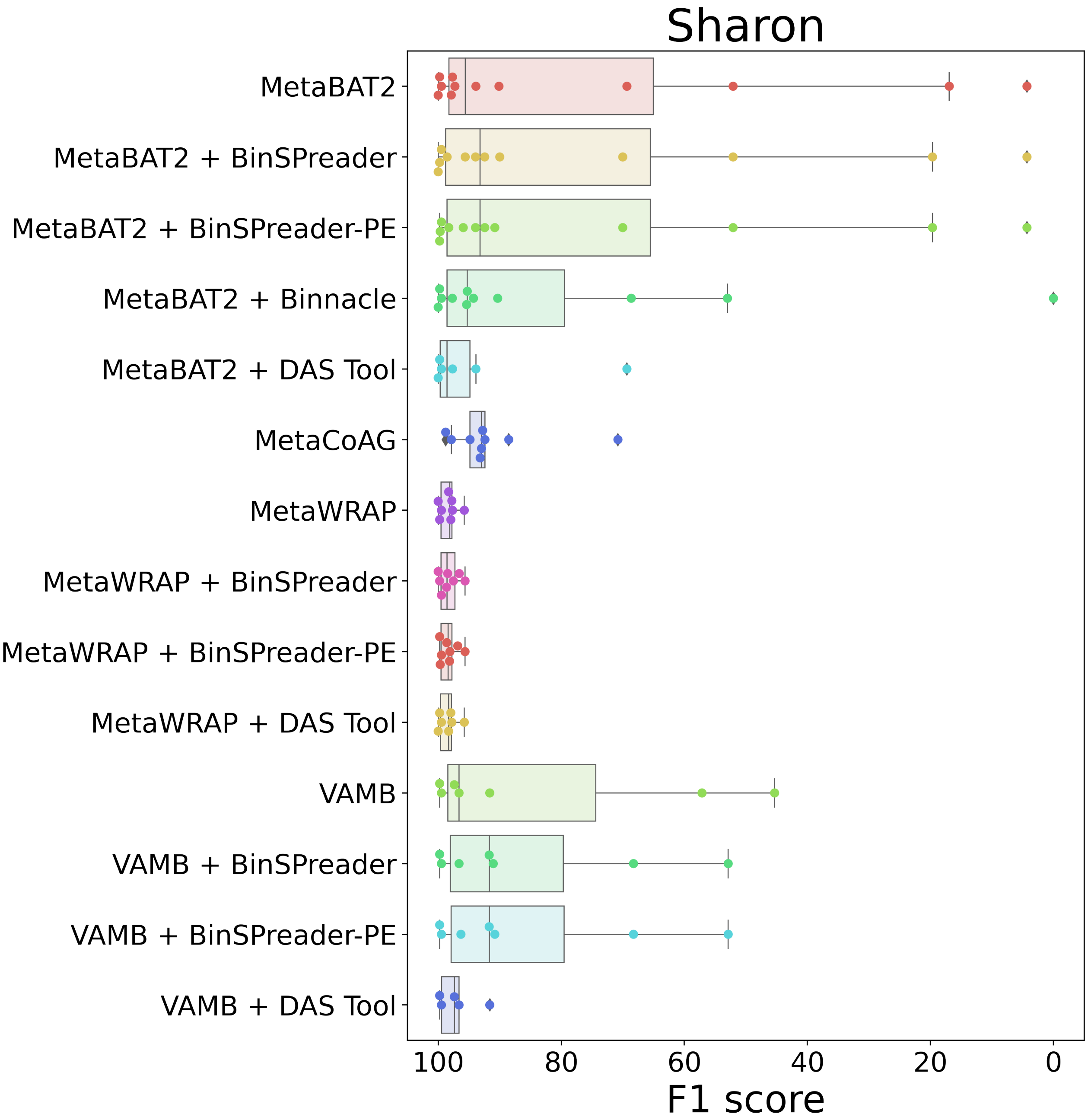

Supplementary Figure 11: F1 score distributions of **Sharon** bins, related to Figure 1.

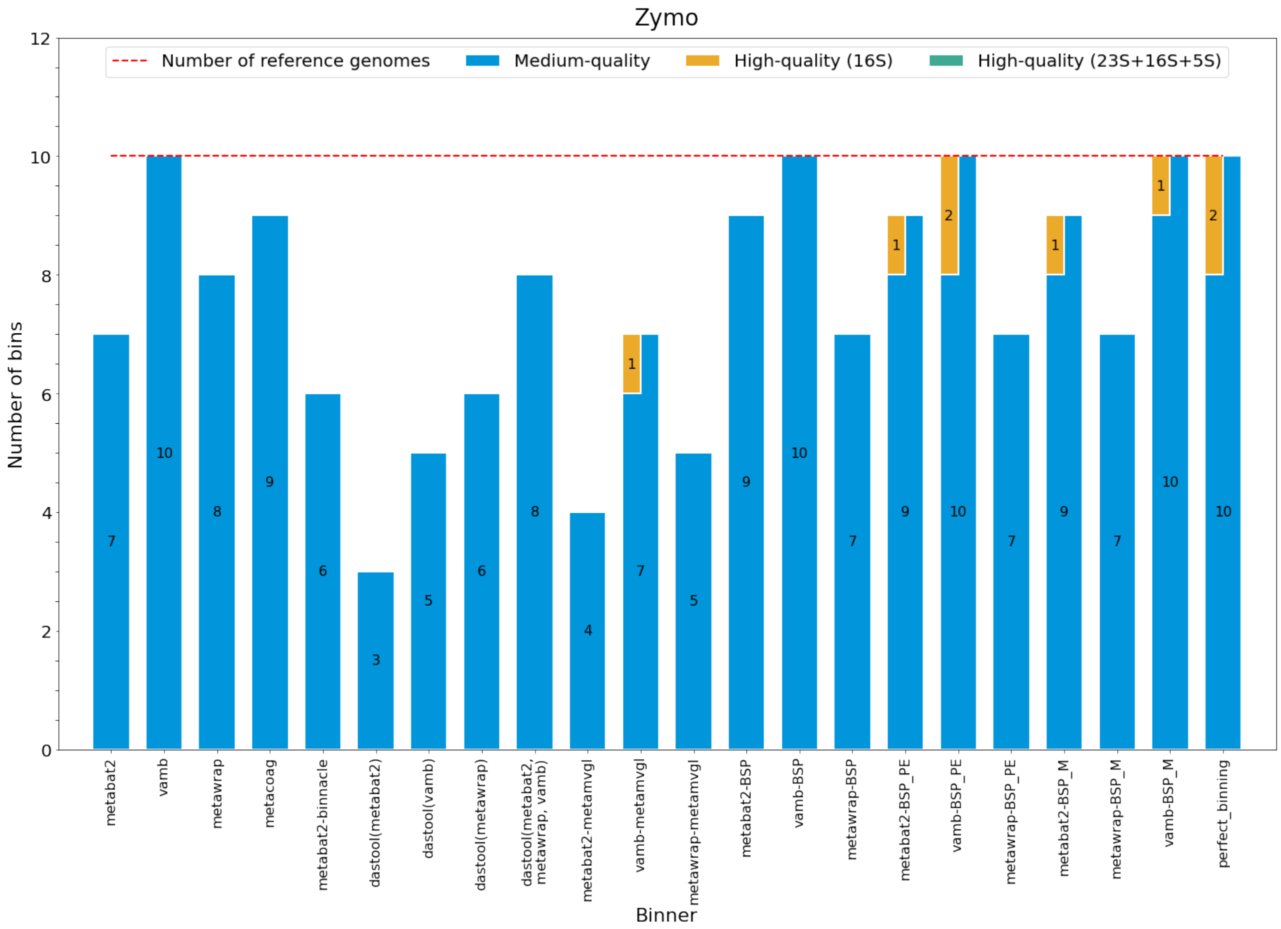

Supplementary Figure 12: MIMAG for **Zymo** dataset. BSP denotes refining with BinSPreader in default mode, BSP_M denotes BinSPreader with multiple binning and BSP_PE denotes BinSPreader with the usage of supplementary paired-end connectivity information, related to Figure 1. perfect_binning – MAGs constructed from assembly having 100% purity and completeness. High-quality MAGs are divided into MAGs containing at least 16S/18S (orange bars) and MAGs with complete set of rRNA (green bars). Dashed line indicates number of reference genomes in dataset.

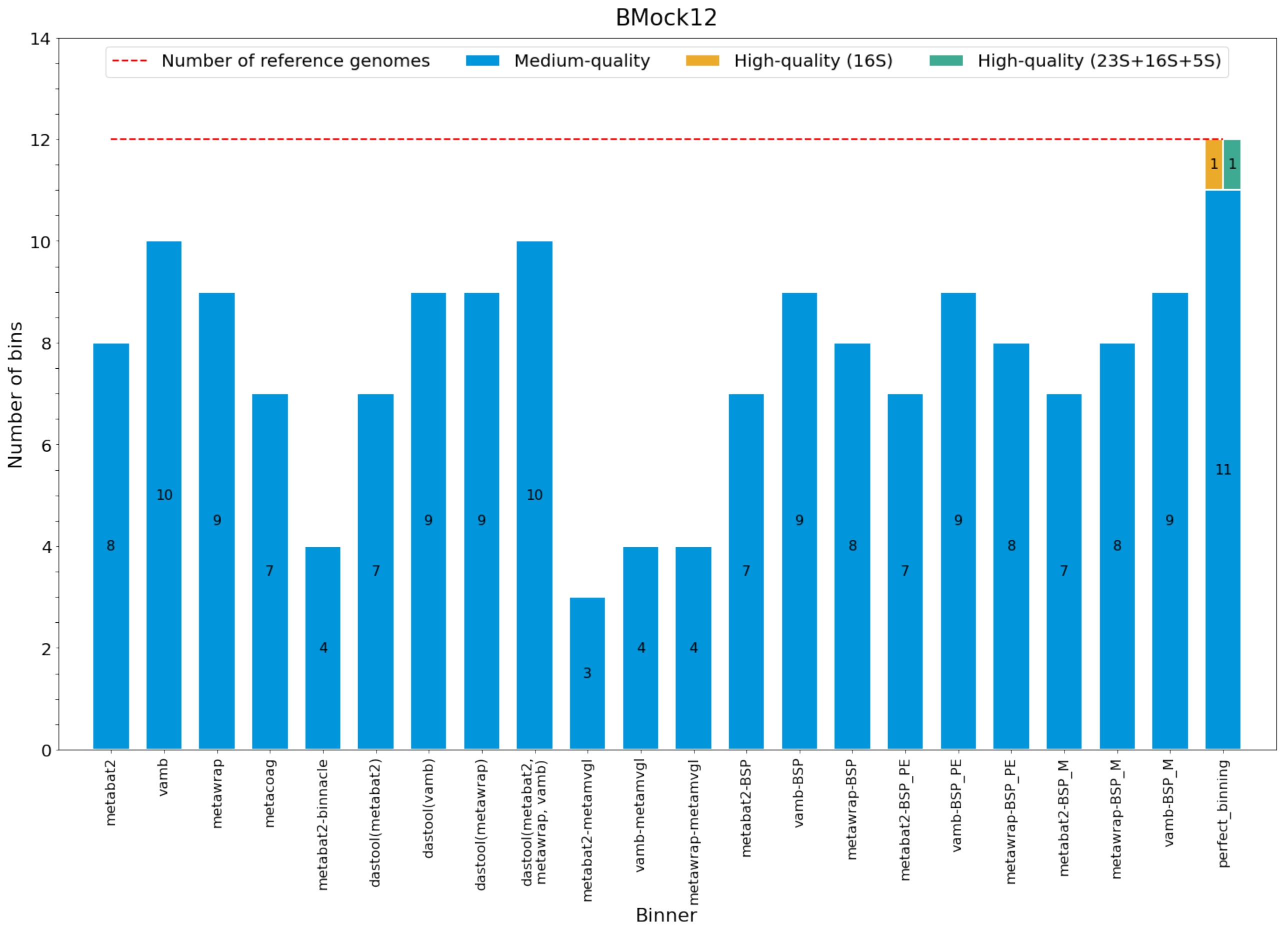

Supplementary Figure 13: MIMAG for **BMock12** dataset. BSP denotes refining with BinSPreader in default mode, BSP_M denotes BinSPreader with multiple binning and BSP_PE denotes BinSPreader with the usage of supplementary paired-end connectivity information, related to Figure 1. perfect_binning – MAGs constructed from assembly having 100% purity and completeness. High-quality MAGs are divided into MAGs containing at least 16S/18S (orange bars) and MAGs with complete set of rRNA (green bars). Dashed line indicates number of reference genomes in dataset.

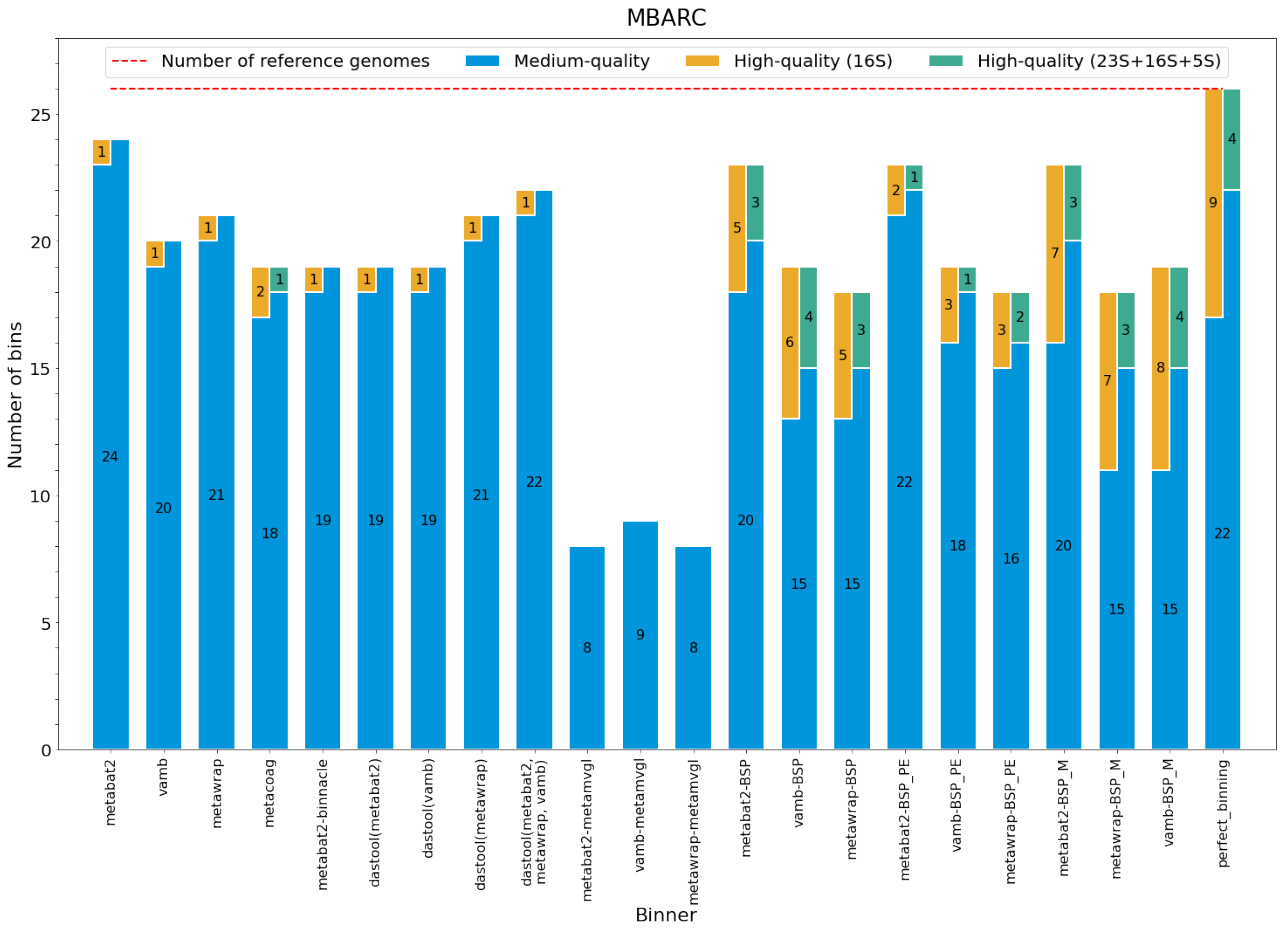

Supplementary Figure 14: MIMAG for **MBARC26** dataset. BSP denotes refining with BinSPreader in default mode, BSP_M denotes BinSPreader with multiple binning and BSP_PE denotes BinSPreader with the usage of supplementary paired-end connectivity information, related to Figure 1. perfect_binning – MAGs constructed from assembly having 100% purity and completeness. High-quality MAGs are divided into MAGs containing at least 16S/18S (orange bars) and MAGs with complete set of rRNA (green bars). Dashed line indicates number of reference genomes in dataset.

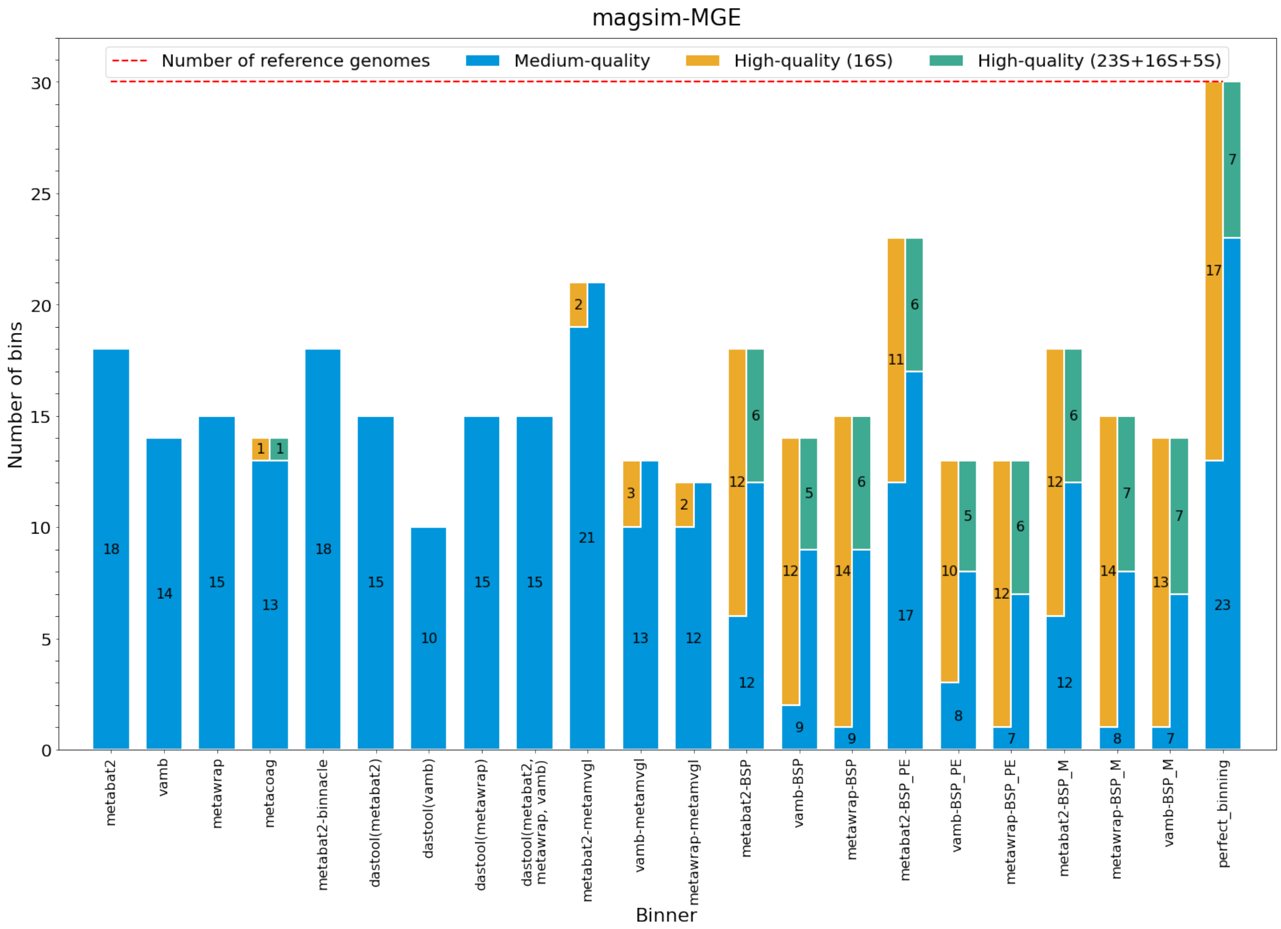

Supplementary Figure 15: MIMAG for **magsim-MGE** dataset. BSP denotes refining with BinSPreader in default mode, BSP_M denotes BinSPreader with multiple binning and BSP_PE denotes BinSPreader with the usage of supplementary paired-end connectivity information, related to Figure 1. perfect_binning – MAGs constructed from assembly having 100% purity and completeness. High-quality MAGs are divided into MAGs containing at least 16S/18S (orange bars) and MAGs with complete set of rRNA (green bars). Dashed line indicates number of reference genomes in dataset.

3 Supplementary Tables

Tool	AC, bp, %	AP, bp, %	F1, bp, %	% binned, by length	% binned, by # seq	# recovered genomes depending on completeness		
						>50%	>70%	>90%
Gold standard	100.0	100.0	100.0	100.0	100.0	30	30	30
DAS Tool + MetaBAT2 + MetaWRAP + VAMB	47.3	**99.8**	64.2	59.0	8.6	15	**15**	12
MetaBAT2	69.2	97.2	80.8	78.5	30.7	18	**15**	12
MetaBAT2 + BinSPreader	72.0	97.0	82.6	81.0	35.0	17	14	12
MetaBAT2 + BinSPreader-PE	**75.8**	96.6	**85.0**	**84.1**	**44.3**	**23**	14	12
MetaBAT2 + Binnacle	66.0	97.7	78.8	78.7	32.5	18	12	9
MetaBAT2 + DAS Tool	47.1	**99.8**	64.0	58.4	7.9	15	**15**	12
MetaBAT2 + METAMVGL	71.7	95.9	82.0	77.4	43.9	21	13	9
MetaCoAG	47.3	97.5	63.7	57.5	8.6	12	12	10
MetaWRAP	47.4	**99.9**	64.3	59.0	8.6	15	**15**	13
MetaWRAP + BinSPreader	50.9	99.1	67.3	62.4	12.2	14	14	**14**
MetaWRAP + BinSPreader-PE	53.0	97.7	68.7	63.6	15.2	13	13	13
MetaWRAP + DAS Tool	47.4	**99.9**	64.3	59.0	8.6	15	**15**	13
MetaWRAP + METAMVGL	49.6	97.1	65.7	59.5	15.4	11	11	10
VAMB	43.5	**99.8**	60.6	52.8	4.6	14	13	8
VAMB + BinSPreader	49.6	98.9	66.1	59.8	10.1	14	13	13
VAMB + BinSPreader-PE	52.2	97.4	67.9	62.1	13.9	13	13	12
VAMB + DAS Tool	30.9	**99.9**	47.1	32.5	2.1	10	10	6
VAMB + METAMVGL	49.6	97.5	65.7	59.3	14.8	13	12	11

Supplementary Table 1: AMBER results for **magsim-MGE** dataset. AC denotes average completeness, AP denotes average purity. The best results for each metrics are highlighted in bold. Related to section Results.

Tool	AC, bp, %	AP, bp, %	F1, bp, %	% binned, bp	% binned, seq	# recovered genomes depending on completeness		
						>50%	>70%	>90%
Gold standard	100.0	100.0	100.0	100.0	100.0	100	100	100
MaxBin2	79.3	90.4	84.5	84.9	85.3	48	48	43
MaxBin2 + BinSPreader	87.0	84.9	85.9	**100.0**	**99.9**	41	41	39
MetaBAT2	13.0	**98.3**	23.0	14.8	0.8	8	4	4
MetaBat2 + BinSPreader	88.4	67.1	76.3	94.3	89.1	8	7	6
MetaCoAG	82.7	91.1	86.7	92.0	90.0	53	52	43
VAMB	91.4	92.0	91.7	95.2	63.8	**66**	**65**	56
VAMB + BinSPreader	**97.1**	91.3	**94.1**	**99.9**	98.9	63	63	**61**

Supplementary Table 2: AMBER results for **simHC+** dataset. AC denotes average completeness, AP denotes average purity. The best results for each metrics are highlighted in bold. Related to section Results.

Tool	AC, bp, %	AP, bp, %	F1, bp, %	% binned, bp	% binned, seq	# recovered genomes depending on completeness		
						>50%	>70%	>90%
Gold standard	100.0	100.0	100.0	100.0	100.0	10	10	10
DAS Tool + MetaBAT2 + MetaWRAP + VAMB	77.9	**100.0**	87.6	50.6	26.5	8	8	7
MetaBAT2	66.2	**100.0**	79.7	91.7	44.4	7	4	3
MetaBAT2 + BinSPreader	78.3	99.4	87.6	**99.8**	99.3	9	6	5
MetaBAT2 + BinSPreader-PE	79.8	**100.0**	88.8	**100.0**	**100.0**	9	6	5
MetaBAT2 + Binnacle	60.6	**100.0**	75.4	70.7	52.7	6	4	3
MetaBAT2 + DAS Tool	35.8	**100.0**	52.7	30.4	16.5	3	3	2
MetaBAT2 + METAMVGL	77.9	88.4	82.8	81.2	89.2	4	4	2
MetaCoAG	74.6	98.6	85.0	50.6	31.4	9	6	5
MetaWRAP	78.8	**100.0**	88.1	42.7	15.9	8	8	8
MetaWRAP + BinSPreader	99.7	95.4	97.5	**99.8**	99.4	7	7	7
MetaWRAP + BinSPreader-PE	**99.9**	95.1	97.5	**100.0**	**100.0**	7	7	7
MetaWRAP + DAS Tool	59.5	**100.0**	74.6	29.7	9.0	6	6	6
MetaWRAP + METAMVGL	66.5	94.3	78.0	80.9	93.3	5	3	1
VAMB	96.5	**100.0**	98.2	95.6	51.2	**10**	**10**	**10**
VAMB + BinSPreader	99.6	**99.8**	**99.7**	**99.8**	99.4	**10**	**10**	**10**
VAMB + BinSPreader-PE	**100.0**	**100.0**	**100.0**	**100.0**	**100.0**	**10**	**10**	**10**
VAMB + DAS Tool	49.1	**100.0**	65.9	23.9	7.7	5	5	5
VAMB + METAMVGL	78.0	94.8	85.6	86.3	94.5	7	5	3

Supplementary Table 3: AMBER results for mock **Zymo** dataset. AC denotes average completeness, AP denotes average purity. The best results for each metrics are highlighted in bold. Related to section Results.

Tool	AC, bp, %	AP, bp, %	F1, bp, %	% binned, bp	% binned, seq	# recovered genomes depending on completeness		
						>50%	>70%	>90%
Gold standard	100.0	100.0	100.0	100.0	100.0	25	25	25
DAS Tool + MetaBAT2 + MetaWRAP + VAMB	88.7	99.2	93.6	89.2	50.4	22	**22**	**20**
MetaBAT2	88.9	98.0	93.2	96.1	62.2	**23**	20	15
MetaBAT2 + BinSPreader	92.1	97.3	94.7	**99.9**	99.3	22	19	17
MetaBAT2 + BinSPreader-PE	91.0	96.2	93.5	**100.0**	**99.7**	20	17	14
MetaBAT2 + Binnacle	82.0	97.6	89.1	96.4	71.1	18	15	10
MetaBAT2 + DAS Tool	74.5	99.6	85.2	73.1	39.0	18	18	14
MetaBAT2 + METAMVGL	67.3	86.5	75.7	66.5	84.9	8	8	6
MetaCoAG	92.6	93.9	93.2	96.5	89.4	17	16	15
MetaWRAP	80.9	**99.8**	89.4	79.7	47.8	21	21	19
MetaWRAP + BinSPreader	**98.9**	92.3	**95.5**	**99.9**	99.3	18	18	17
MetaWRAP + BinSPreader-PE	96.3	92.3	94.2	**100.0**	**99.7**	18	18	17
MetaWRAP + DAS Tool	80.9	**99.8**	89.4	79.7	47.8	21	21	19
MetaWRAP + METAMVGL	69.5	90.1	78.5	69.8	87.4	8	8	5
VAMB	91.2	95.1	93.1	92.6	48.1	20	20	16
VAMB + BinSPreader	96.1	92.9	94.5	**99.9**	99.3	19	19	18
VAMB + BinSPreader-PE	95.3	92.7	94.0	**100.0**	**99.7**	19	19	17
VAMB + DAS Tool	72.8	**99.9**	84.2	71.9	36.1	19	19	16
VAMB + METAMVGL	65.8	89.4	75.8	67.5	80.4	9	8	6

Supplementary Table 4: AMBER results for **MBARC26** dataset. AC denotes average completeness, AP denotes average purity. The best results for each metrics are highlighted in bold. Related to section Results.

Tool	AC, bp, %	AP, bp, %	F1, bp, %	% binned, bp	% binned, seq	# recovered genomes depending on completeness		
						>50%	>70%	>90%
Gold standard	100.0	100.0	100.0	100.0	100.0	11	11	11
DAS Tool + MetaBAT2 + MetaWRAP + VAMB	77.9	**98.4**	86.9	86.5	37.6	**9**	**9**	5
MetaBAT2	66.9	97.7	79.4	91.8	34.5	7	7	2
MetaBAT2 + BinSPreader	84.2	95.5	89.5	**99.8**	97.1	6	6	2
MetaBAT2 + BinSPreader-PE	83.6	95.2	89.0	**100.0**	**98.8**	6	6	2
MetaBAT2 + Binnacle	49.5	94.8	65.1	87.5	53.4	3	2	1
MetaBAT2 + DAS Tool	57.4	96.3	71.9	71.8	21.8	6	6	2
MetaBAT2 + METAMVGL	74.7	85.1	79.5	71.0	87.5	3	2	1
MetaCoAG	75.1	88.6	81.3	94.8	65.8	5	5	4
MetaWRAP	79.3	96.5	87.1	92.0	40.7	8	8	6
MetaWRAP + BinSPreader	**96.4**	92.7	**94.6**	**99.8**	97.1	8	8	**7**
MetaWRAP + BinSPreader-PE	95.2	91.1	93.1	**100.0**	**98.8**	8	8	6
MetaWRAP + DAS Tool	79.3	96.5	87.1	92.0	40.7	8	8	6
MetaWRAP + METAMVGL	75.6	87.9	81.3	71.1	89.8	4	4	2
VAMB	77.9	98.1	86.8	91.0	31.7	8	8	3
VAMB + BinSPreader	95.2	93.4	94.3	**99.8**	97.1	6	6	5
VAMB + BinSPreader-PE	95.2	93.6	94.4	**100.0**	**98.8**	6	6	4
VAMB + DAS Tool	69.9	97.6	81.5	79.4	25.3	7	7	3
VAMB + METAMVGL	70.1	88.9	78.4	65.6	88.9	4	3	2

Supplementary Table 5: AMBER results for **BMock12** dataset. AC denotes average completeness, AP denotes average purity. The best results for each metrics are highlighted in bold. Related to section Results.

Tool	AC, bp, %	AP, bp, %	F1, bp, %	% binned, bp	% binned, seq	# recovered genomes depending on completeness		
						>50%	>70%	>90%
Gold standard	100.0	100.0	100.0	100.0	100.0	10	10	10
bin3C	96.1	**96.7**	96.4	88.3	59.5	7	7	6
bin3C + BinSPreader	99.6	96.5	98.0	**100.0**	99.7	**7**	**7**	**7**
bin3C + BinSPreader-PE	**99.7**	96.6	**98.2**	**100.0**	**100.0**	**7**	**7**	**7**

Supplementary Table 6: AMBER results for **Zymo** dataset for bin3C binning. AC denotes average completeness, AP denotes average purity. The best results for each metrics are highlighted in bold. Related to section Results.

Tool	# bins	AC %	AP %	F1 %	# recovered genomes (90% completeness)
MetaBAT2	50	58.5	99.0	64.7	15
MetaBAT2 + BinSPreader	50	59.8	97.3	64.9	16
MetaBAT2 + BinSPreader-HiC	50	69.1	92.7	69.6	15
MetaBAT2 + BinSPreader-PE	50	60.1	97.3	65.4	16
MetaBAT2 + Binnacle	57	51.0	99.2	57.2	15
MetaBAT2 + DAS Tool	23	87.7	99.4	92.3	13
MetaBAT2 + METAMVGL	46	46.2	97.1	53.8	4
MetaCoAG	37	82.5	95.4	86.2	22
MetaWRAP	31	94.2	99.4	96.5	**26**
MetaWRAP + BinSPreader	31	94.7	95.7	94.9	23
MetaWRAP + BinSPreader-HiC	31	97.3	88.5	91.1	19
MetaWRAP + BinSPreader-PE	31	94.8	95.0	94.7	21
MetaWRAP + DAS Tool	28	94.7	99.4	96.8	24
MetaWRAP + METAMVGL	31	72.1	95.7	80.2	7
VAMB	25	86.3	99.5	90.7	17
VAMB + BinSPreader	25	91.3	93.4	90.5	13
VAMB + BinSPreader-HiC	25	**98.9**	78.2	84.1	14
VAMB + BinSPreader-PE	25	91.4	91.4	89.4	12
VAMB + DAS Tool	19	94.3	99.5	96.5	17
VAMB + METAMVGL	25	69.2	93.0	75.6	2
bin3C	168	17.9	**99.8**	18.6	**26**
bin3C + BinSPreader	168	18.3	99.4	18.8	**26**
bin3C + BinSPreader-HiC	168	21.9	98.1	22.0	24
bin3C + BinSPreader-PE	168	18.4	99.4	18.8	**26**
bin3C + DAS Tool	29	96.4	99.2	**97.7**	**26**
bin3C + METAMVGL	157	15.1	99.2	17.0	6

Supplementary Table 7: CheckM results for **IC9** dataset. Related to section Results.

Tool	# bins	AC %	AP %	F1 %	# recovered genomes (90% completeness)
MetaBAT2	12	72.2	97.7	76.6	6
MetaBAT2 + BinSPreader	12	73.1	95.6	76.3	5
MetaBAT2 + BinSPreader-PE	12	73.1	95.7	76.4	5
MetaBAT2 + Binnacle	11	77.4	96.9	81.3	6
MetaBAT2 + DAS Tool	6	90.5	96.9	93.4	4
MetaCoAG	9	88.9	94.7	91.3	3
MetaWRAP	8	97.5	**99.2**	**98.3**	**8**
MetaWRAP + BinSPreader	8	**98.2**	98.3	**98.3**	**8**
MetaWRAP + BinSPreader-PE	8	**98.3**	98.3	**98.3**	**8**
MetaWRAP + DAS Tool	7	97.8	**99.1**	**98.4**	7
VAMB	7	85.7	90.1	83.9	4
VAMB + BinSPreader	7	90.2	85.5	85.7	3
VAMB + BinSPreader-PE	7	90.2	85.3	85.6	3
VAMB + DAS Tool	5	95.6	98.7	97.0	4

Supplementary Table 8: CheckM results for **Sharon** dataset. Related to section Results.

**Dataset/Binner**	**MBARC**	**BMock**	**Zymo**	**magsim-MGE**
**MetaBAT2**	32	11	11	11
**VAMB**	31	13	**12**	8
**metaWRAP**	33	11	**12**	12
**MetaCoAG**	42	12	**12**	12
**MetaBAT2-Binnacle**	31	12	**12**	13
**DAS TOOL (MetaBAT2)**	26	5	5	11
**DAS TOOL (VAMB)**	28	8	8	8
**DAS TOOL (metaWRAP)**	33	11	8	12
**DAS TOOL (MetaBAT2, metaWRAP, VAMB)**	30	11	10	12
**MetaBAT2-METAMVGL**	35	12	10	**17**
**VAMB-METAMVGL**	36	14	8	14
**metaWRAP-METAMVGL**	36	14	6	16
**MetaBAT2-BinSPreader**	**44**	**17**	**12**	13
**VAMB-BinSPreader**	**44**	**17**	**12**	11
**metaWRAP-BinSPreader**	**44**	**17**	**12**	14
**MetaBAT2-BinSPreader-PE**	**44**	**17**	**12**	**17**
**VAMB-BinSPreader-PE**	**44**	**17**	**12**	13
**metaWRAP-BinSPreader-PE**	**44**	**17**	**12**	16
**MetaBAT2-BinSPreader-Multiple**	**44**	**17**	**12**	13
**VAMB-BinSPreader-Multiple**	**44**	**17**	**12**	11
**metaWRAP-BinSPreader-Multiple**	**44**	**17**	**12**	14
**Assembly**	44	17	12	23
**Reference genomes**	54	19	12	32

Supplementary Table 9: Numbers of recovered CRISPRs for mock metagenomes and **magsim-MGE** dataset. BinSPreader-PE denotes refining mode of BinSPreader utilizing additional paired-end links, and BinSPreader-Multiple denotes refining mode with multiple binning of contigs (but without paired-end data). Best results are highlighted in bold. Related to section Results.

**Dataset/Binner**	**MBARC**	**BMock**	**Zymo**	**magsim-MGE**
**MetaBAT2**	130	**8**	122	115
**VAMB**	104	**8**	129	76
**metaWRAP**	54	**8**	130	102
**MetaCoAG**	**138**	6	133	106
**MetaBAT2-Binnacle**	135	7	129	117
**DAS TOOL (MetaBAT2)**	17	5	29	101
**DAS TOOL (VAMB)**	22	7	36	20
**DAS TOOL (metaWRAP)**	54	**8**	45	102
**DAS TOOL (MetaBAT2, metaWRAP, VAMB)**	55	**8**	81	102
**MetaBAT2-METAMVGL**	103	5	95	108
**VAMB-METAMVGL**	66	5	111	97
**metaWRAP-METAMVGL**	121	5	63	92
**MetaBAT2-BinSPreader**	**139**	**8**	133	135
**VAMB-BinSPreader**	**139**	**8**	133	112
**metaWRAP-BinSPreader**	**139**	**8**	133	121
**MetaBAT2-BinSPreader-PE**	**139**	**8**	**138**	**138**
**VAMB-BinSPreader-PE**	**139**	**8**	**138**	124
**metaWRAP-BinSPreader-PE**	**139**	**8**	**138**	124
**MetaBAT2-BinSPreader-Multiple**	**139**	**8**	133	135
**VAMB-BinSPreader-Multiple**	**139**	**8**	133	112
**metaWRAP-BinSPreader-Multiple**	**139**	**8**	133	121
**Assembly**	139	8	138	145
**Reference genomes**	153	11	182	220

Supplementary Table 10: Numbers of recovered AMR genes for mock metagenomes and **magsim-MGE** dataset. BinSPreader-PE denotes refining mode of BinSPreader utilizing paired-end links, and BinSPreader-Multiple denotes refining mode with multiple binning of contigs (but without paired-end data). Best results are highlighted in bold.

Binner	Genes recovered
**MetaBAT2**	70
**VAMB**	54
**metaWRAP**	70
**MetaCoAG**	134
**Bin3C**	127
**MetaBAT2-Binnacle**	73
**DAS TOOL (MetaBAT2)**	51
**DAS TOOL (VAMB)**	49
**DAS TOOL (metaWRAP)**	65
**DAS TOOL (Bin3C)**	115
**MetaBAT2-METAMVGL**	139
**VAMB-METAMVGL**	131
**metaWRAP-METAMVGL**	136
**Bin3C-METAMVGL**	146
**MetaBAT2-BinSPreader**	160
**VAMB-BinSPreader**	145
**metaWRAP-BinSPreader**	156
**Bin3C-BinSPreader**	165
**MetaBAT2-BinSPreader-PE**	161
**VAMB-BinSPreader-PE**	146
**metaWRAP-BinSPreader-PE**	157
**Bin3C-BinSPreader-PE**	166
**MetaBAT2-BinSPreader-HiC**	**191**
**metaWRAP-BinSPreader-HiC**	**191**
**VAMB-BinSPreader-HiC**	**191**
**Bin3C-BinSPreader-HiC**	**191**
**Assembly**	300

Supplementary Table 11: Numbers of recovered AMR (AntiMicrobial Resistance) genes for **IC9** dataset. BinSPreader-PE denotes refining mode of BinSPreader utilizing paired-end links, and BinSPreader-HiC denotes refining mode utilizing Hi-C links. Related to section Results.

Zymo		
**Binner**	**GF >50%**	**GF >90%**
**MetaBAT2**	0	0
**VAMB**	0	0
**metaWRAP**	0	0
**MetaCoAG**	2	1
**MetaBAT2-Binnacle**	0	0
**DAS TOOL (MetaBAT2)**	0	0
**DAS TOOL (VAMB)**	0	0
**DAS TOOL (metaWRAP)**	0	0
**DAS TOOL (MetaBAT2, metaWRAP, VAMB)**	0	0
**MetaBAT2-METAMVGL**	1	1
**VAMB-METAMVGL**	1	1
**metaWRAP-METAMVGL**	2	1
**MetaBAT2-BinSPreader**	4	1
**VAMB-BinSPreader**	4	1
**metaWRAP-BinSPreader**	4	1
**MetaBAT2-BinSPreader-PE**	**5**	**2**
**VAMB-BinSPreader-PE**	**5**	**2**
**metaWRAP-BinSPreader-PE**	**5**	**2**
**MetaBAT2-BinSPreader-Multiple**	4	1
**metaWRAP-BinSPreader-Multiple**	4	1
**VAMB-BinSPreader-Multiple**	4	1
**Assembly**	5	2

Supplementary Table 12: Number of 16S/18S rRNA genes depending on their genome fraction (GF) threshold on **Zymo** dataset. The value of GF indicates the length of the assembled gene in relation to full gene. BinSPreader-PE denotes refining mode of BinSPreader utilizing paired-end links, and BinSPreader-Multiple denotes refining mode with multiple binning of contigs. Related to section Results.

magsim-MGE		
**Binner**	**GF >50%**	**GF >90%**
**MetaBAT2**	1	1
**VAMB**	1	1
**metaWRAP**	2	2
**MetaCoAG**	7	6
**MetaBAT2-Binnacle**	2	2
**DAS TOOL (MetaBAT2)**	1	1
**DAS TOOL (VAMB)**	1	1
**DAS TOOL (metaWRAP)**	2	2
**DAS TOOL (MetaBAT2, metaWRAP, VAMB)**	2	2
**MetaBAT2-METAMVGL**	8	5
**VAMB-METAMVGL**	8	4
**metaWRAP-METAMVGL**	8	4
**MetaBAT2-BinSPreader**	**20**	**17**
**VAMB-BinSPreader**	18	16
**metaWRAP-BinSPreader**	18	16
**MetaBAT2-BinSPreader-PE**	**20**	**17**
**VAMB-BinSPreader-PE**	19	**17**
**metaWRAP-BinSPreader-PE**	19	**17**
**MetaBAT2-BinSPreader-Multiple**	**20**	**17**
**metaWRAP-BinSPreader-Multiple**	18	16
**VAMB-BinSPreader-Multiple**	18	16
**Assembly**	23	18

Supplementary Table 13: Number of 16S/18S rRNA genes depending on their genome fraction (GF) threshold on **magsim-MGE** dataset. The value of GF indicates the length of the assembled gene in relation to full gene. BinSPreader-PE denotes refining mode of BinSPreader utilizing paired-end links, and BinSPreader-Multiple denotes refining mode with multiple binning of contigs. Related to section Results.

BMock12		
**Binner**	**GF >50%**	**GF >90%**
**MetaBAT2**	0	0
**VAMB**	0	0
**metaWRAP**	0	0
**MetaCoAG**	3	**1**
**MetaBAT2-Binnacle**	2	0
**DAS TOOL (MetaBAT2)**	0	0
**DAS TOOL (VAMB)**	0	0
**DAS TOOL (metaWRAP)**	0	0
**DAS TOOL (MetaBAT2, metaWRAP, VAMB)**	0	0
**MetaBAT2-METAMVGL**	0	0
**VAMB-METAMVGL**	0	0
**metaWRAP-METAMVGL**	0	0
**MetaBAT2-BinSPreader**	**4**	**1**
**VAMB-BinSPreader**	**4**	**1**
**metaWRAP-BinSPreader**	**4**	**1**
**MetaBAT2-BinSPreader-PE**	**4**	**1**
**VAMB-BinSPreader-PE**	**4**	**1**
**metaWRAP-BinSPreader-PE**	**4**	**1**
**MetaBAT2-BinSPreader-Multiple**	**4**	**1**
**metaWRAP-BinSPreader-Multiple**	**4**	**1**
**VAMB-BinSPreader-Multiple**	**4**	**1**
**Assembly**	4	1

Supplementary Table 14: Number of 16S/18S rRNA genes depending on their genome fraction (GF) threshold on **BMock12** dataset. The value of GF indicates the length of the assembled gene in relation to full gene. BinSPreader-PE denotes refining mode of BinSPreader utilizing paired-end links, and BinSPreader-Multiple denotes refining mode with multiple binning of contigs. Related to section Results.

MBARC26		
**Binner**	**GF >50%**	**GF >90%**
**MetaBAT2**	2	1
**VAMB**	3	1
**metaWRAP**	3	1
**MetaCoAG**	12	**9**
**MetaBAT2-Binnacle**	7	3
**DAS TOOL (MetaBAT2)**	2	1
**DAS TOOL (VAMB)**	3	1
**DAS TOOL (metaWRAP)**	3	1
**DAS TOOL (MetaBAT2, metaWRAP, VAMB)**	3	1
**MetaBAT2-METAMVGL**	0	0
**VAMB-METAMVGL**	0	0
**metaWRAP-METAMVGL**	0	0
**MetaBAT2-BinSPreader**	**16**	**9**
**VAMB-BinSPreader**	**16**	**9**
**metaWRAP-BinSPreader**	**16**	**9**
**MetaBAT2-BinSPreader-PE**	**16**	**9**
**VAMB-BinSPreader-PE**	**16**	**9**
**metaWRAP-BinSPreader-PE**	**16**	**9**
**MetaBAT2-BinSPreader-Multiple**	**16**	**9**
**metaWRAP-BinSPreader-Multiple**	**16**	**9**
**VAMB-BinSPreader-Multiple**	**16**	**9**
**Assembly**	16	9

Supplementary Table 15: Number of 16S/18S rRNA genes depending on their genome fraction (GF) threshold on **MBARC26** dataset. The value of GF indicates the length of the assembled gene in relation to full gene. BinSPreader-PE denotes refining mode of BinSPreader utilizing paired-end links, and BinSPreader-Multiple denotes refining mode with multiple binning of contigs. Related to section Results.

Tool	16S rRNA		23S rRNA		5S rRNA		
	>50%	>80%	>50%	>80%	>50%	>80%	Total rRNA
Assembly	7	3	12	6	54	47	73
MetaBAT2	0	0	0	0	7	6	7
MetaBAT2 + BinSPreader	**7**	**3**	**12**	**6**	49	40	68
MetaBAT2 + BinSPreader-HiC	**7**	**3**	**12**	**6**	**52**	**44**	**71**
MetaBAT2 + BinSPreader-PE	**7**	**3**	**12**	**6**	49	40	68
MetaBAT2 + Binnacle	0	0	0	0	7	7	7
MetaBAT2 + DAS Tool	0	0	0	0	5	4	5
MetaBAT2 + METAMVGL	1	0	3	1	28	23	32
MetaCoAG	5	**3**	10	5	21	19	36
MetaWRAP	0	0	0	0	8	8	8
MetaWRAP + BinSPreader	**7**	**3**	**12**	**6**	48	40	67
MetaWRAP + BinSPreader-HiC	**7**	**3**	**12**	**6**	**52**	**44**	**71**
MetaWRAP + BinSPreader-PE	**7**	**3**	**12**	**6**	48	40	67
MetaWRAP + DAS Tool	0	0	0	0	8	8	8
MetaWRAP + METAMVGL	0	0	3	1	26	21	29
VAMB	1	1	5	3	6	6	12
VAMB + BinSPreader	**7**	**3**	11	5	47	39	65
VAMB + BinSPreader-HiC	**7**	**3**	**12**	**6**	51	**44**	70
VAMB + BinSPreader-PE	**7**	**3**	**12**	**6**	47	40	66
VAMB + DAS Tool	1	1	5	3	5	5	11
VAMB + METAMVGL	0	0	3	0	22	18	25
bin3C	6	**3**	**12**	**6**	18	16	36
bin3C + BinSPreader	**7**	**3**	**12**	**6**	49	40	68
bin3C + BinSPreader-HiC	**7**	**3**	**12**	**6**	**52**	**44**	**71**
bin3C + BinSPreader-PE	**7**	**3**	**12**	**6**	49	40	68
bin3C + DAS Tool	5	2	8	4	15	14	28
bin3C + METAMVGL	0	0	3	1	21	17	24

Supplementary Table 16: Number of recovered rRNA genes in bins of the **IC9** dataset depending on their completeness. The best results are highlighted in bold. Related to section Results.

Tool	16S rRNA		23S rRNA		5S rRNA		Total rRNA
	>50%	>80%	>50%	>80%	>50%	>80%	
Assembly	7	1	6	4	16	14	29
MetaBAT2	0	0	0	0	1	1	1
MetaBAT2 + BinSPreader	3	**1**	3	**3**	**12**	**10**	18
MetaBAT2 + BinSPreader-PE	**4**	**1**	**4**	**3**	**12**	**10**	**20**
MetaBAT2 + Binnacle	1	0	0	0	1	1	2
MetaBAT2 + DAS Tool	0	0	0	0	1	1	1
MetaCoAG	3	0	1	1	2	2	6
MetaWRAP	1	0	0	0	1	1	2
MetaWRAP + BinSPreader	3	**1**	3	**3**	8	6	14
MetaWRAP + BinSPreader-PE	**4**	**1**	**4**	**3**	**12**	**10**	**20**
VAMB	1	**1**	1	1	2	2	4
VAMB + BinSPreader	3	**1**	3	**3**	8	6	14
VAMB + BinSPreader-PE	**4**	**1**	**4**	**3**	**12**	**10**	**20**
VAMB + DAS Tool	1	**1**	1	1	2	2	4

Supplementary Table 17: Number of recovered rRNA genes in bins of the **Sharon** dataset depending on their completeness. The best results for each metrics are highlighted in bold. Related to section Results.
